# Clinical manifestations and immune response to tuberculosis

**DOI:** 10.1007/s11274-023-03636-x

**Published:** 2023-05-24

**Authors:** Mary Lilián Carabalí-Isajar, Oscar Hernán Rodríguez-Bejarano, Tatiana Amado, Manuel Alfonso Patarroyo, María Alejandra Izquierdo, Juan Ricardo Lutz, Marisol Ocampo

**Affiliations:** 1 Fundación Instituto de Inmunología de Colombia (FIDIC), Carrera 50#26–20, 111321 Bogotá, Colombia; 2 Biomedical and Biological Sciences Programme, Universidad del Rosario, Carrera 24#63C-69, 111221 Bogotá, Colombia; 3 Health Sciences Faculty, Universidad de Ciencias Aplicadas y Ambientales (UDCA), Calle 222#55-37, 111166 Bogotá, Colombia; 4 Faculty of Medicine, Universidad Nacional de Colombia, Carrera 45#26-85, 111321 Bogotá, Colombia; 5 Medicine Department, Hospital Universitario Mayor Mederi, Calle 24 # 29-45, 111411 Bogotá, Colombia; 6 Universidad Distrital Francisco José de Caldas, Carrera 3#26A–40, 110311 Bogotá, Colombia

**Keywords:** Tuberculosis, Immune response, *Mycobacterium tuberculosis*, Clinical manifestations

## Abstract

Tuberculosis is a far-reaching, high-impact disease. It is among the top ten causes of death worldwide caused by a single infectious agent; 1.6 million tuberculosis-related deaths were reported in 2021 and it has been estimated that a third of the world’s population are carriers of the tuberculosis bacillus but do not develop active disease. Several authors have attributed this to hosts’ differential immune response in which cellular and humoral components are involved, along with cytokines and chemokines. Ascertaining the relationship between TB development’s clinical manifestations and an immune response should increase understanding of tuberculosis pathophysiological and immunological mechanisms and correlating such material with protection against *Mycobacterium tuberculosis*. Tuberculosis continues to be a major public health problem globally. Mortality rates have not decreased significantly; rather, they are increasing. This review has thus been aimed at deepening knowledge regarding tuberculosis by examining published material related to an immune response against *Mycobacterium tuberculosis*, mycobacterial evasion mechanisms regarding such response and the relationship between pulmonary and extrapulmonary clinical manifestations induced by this bacterium which are related to inflammation associated with tuberculosis dissemination through different routes.

## Introduction

Humanity has been scourged by tuberculosis (TB) for centuries; it is an infectious bacterial disease and has become positioned as one of the main causes of mortality worldwide. This infection is mainly caused by the *Mycobacterium tuberculosis* (*Mtb*) bacillus which is found in the MTB complex; it is principally transmitted through aerosols that are expelled from a person having active TB. Efforts at slowing the disease’s progression have involved studies in fields related to epidemiology, risk factors, immune response, TB pathophysiology, new diagnostic and therapeutic tools for all forms of infection and the disease itself (Furin et al. 2019).

TB has primarily been considered a pulmonary disease by the classical approach; however, *Mtb* can spread from an initial source of infection via different routes and can affect almost all the body’s organs. A 2021 estimate showed that around 10.6 million people had become ill with TB, representing an increase from 10.1 million in 2020; incidence rate increased by 3.6% between 2020 and 2021 and 1.4 million cases occurred. Regarding TB-related deaths, 57% of cases were men, 33% women and 11% children (World Health Organization 2022).

Once inhaled, *Mtb* is faced with a first line of immune defence consisting of airway epithelial cells (AEC) and phagocytic cells (neutrophils (N), monocytes (M) and dendritic cells (DC). Infection does not occur if such first line of defence succeeds in rapidly eliminating *Mtb*, otherwise phagocytes become infected and *Mtb* reproduces within these cells, initially causing few if any clinical manifestations (de Martino et al. 2019).

Most *Mtb*-infected people control the infection without intervention, such clinically inactive infection stage being referred to as latent TB; a small percentage of those having the latent stage will progress to an active TB stage years or decades after primary infection. A broad spectrum of presentation can result from *Mtb* infection, ranging from subclinical infection to mild, moderate or severe active clinical infection (Behr et al. 2019).

The bacilli undergo haematogenous and lymphatic dissemination during primary infection, affecting the pulmonary and mediastinal hilar lymph nodes; they can reach different organs through lymphohematogenous dissemination, resulting in extrapulmonary TB (EPTB) during primary infection or during TB reactivation later in life. EPTB can involve any host body organ, the lymph nodes being the most common site; however, pleural, neurological, osteoarticular and/or genitourinary involvement have also been described, each having a particular clinical presentation associated with an acute or chronic inflammatory response triggered by the immune system to destroy *Mtb* (Fogel 2015).

**Fig. 1:**
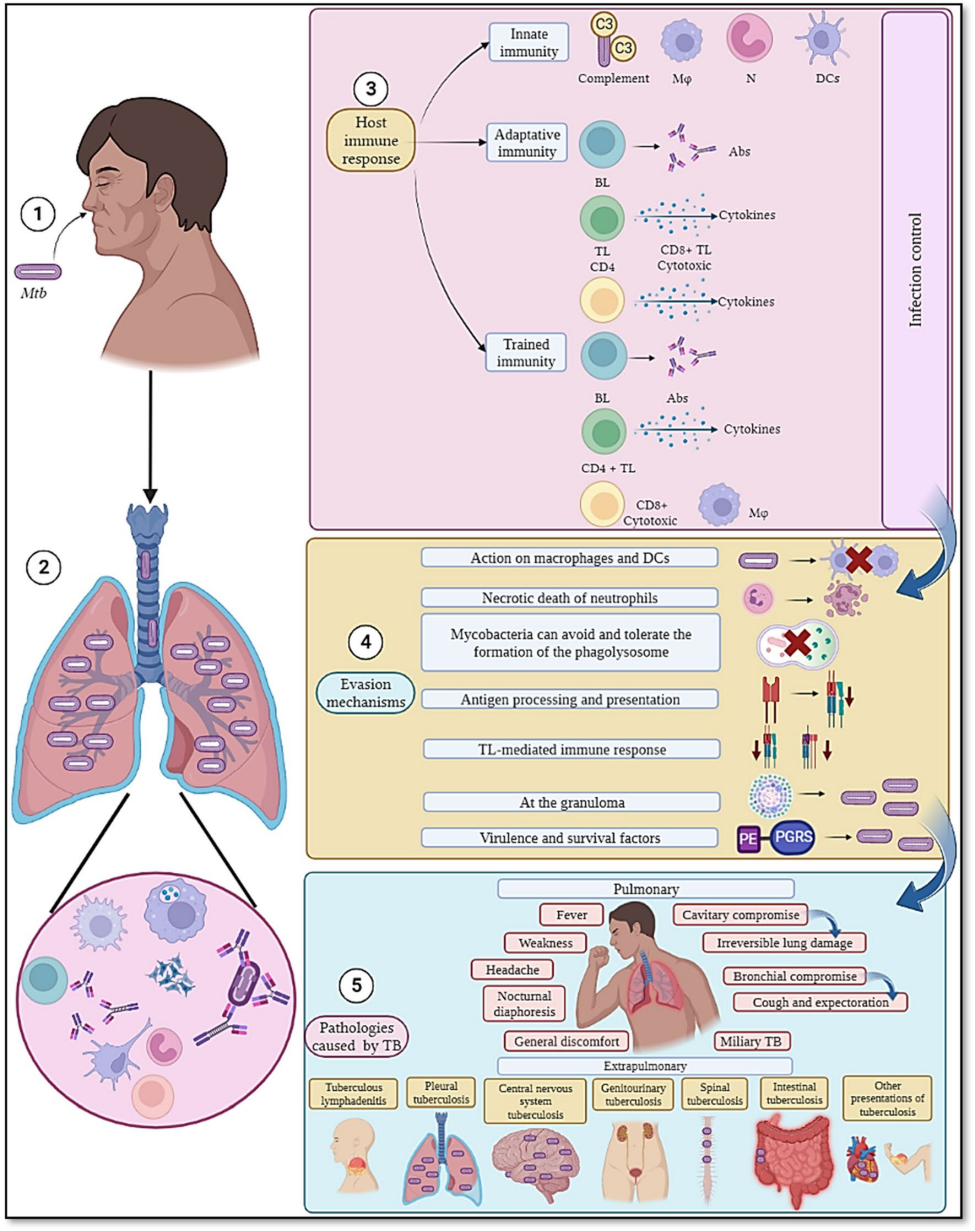
Immune response to and clinical manifestations of tuberculosis. Once the mycobacteria enter a host’s body by airway **①** they are perceived by its immune system **②** and this can lead to three outcomes. **③** An innate immune response may become overwhelmed; complement factors can bind to mycobacteria and create a pore leading to microorganism lysis while cells such as neutrophils (N) macrophages (Mφ) and dendritic cells (DCs) try to control the infection by engulfing the mycobacteria and, in turn, antigen presentation occurs. An adaptive immune response is thus induced during which *Mtb*-specific antibodies (Ab) are produced, having different effector functions targeting the microorganism, along with cytokine production by B-lymphocytes (BL). Such Ab production is often mediated by CD4 + T-lymphocytes (TL), which convert BL into Ab-producing plasma cells. CD4 + TL cells also help eliminate mycobacteria intracellularly in infected cells, whilst cytotoxic cells (CD8 + TL) directly destroy cells carrying the tubercle bacillus. The role of trained immunity is worth noting; such a concept proposes that immune system cells and Abs have previously been trained to attack pathogens, whether they are similar or different to those that gave rise to the initial immune reaction. **④** However, *Mtb* has developed different evasion mechanisms against a host’s immune response by manipulating cells such as Mφ where it can establish a niche and multiply, in addition to manipulating alveolar epithelial cells (AEC) and neutrophils (N) leading to necrosis. It also avoids antigen processing and presentation for which phagolysosome formation is essential and during which *Mtb* must be destroyed, however, *Mtb* avoids or tolerates it. The lack of antigen presentation affects a lymphocyte-mediated immune response, mainly a T-mediated one. Granuloma formation is designed to contain and eliminate *Mtb*; this is used by the pathogen to remain in a state of latency while waiting to become able to colonise other host cells. *Mtb* has genes that encode PE-PGRS proteins thereby enabling it to survive in a host and favourably immunomodulate its response. **⑤** Thus, if a host’s immune response is deficient and/or *Mtb* can correctly evade it, such infection can result in active tuberculosis, i.e. pulmonary TB causing the greatest amount of cases worldwide and extrapulmonary TB. Figure created using Biorender.com.

This review discusses the innate and adaptive immune response triggered by *Mtb*, its evasion mechanisms used in the attempt to survive host immune system defence and the clinical manifestations of TB related to pulmonary and extrapulmonary involvement, summarised in Fig. 1.

### The immune response: how does the immune system react against an unexpected attack by mycobacteria?

#### Innate immune response

**Fig. 2:**
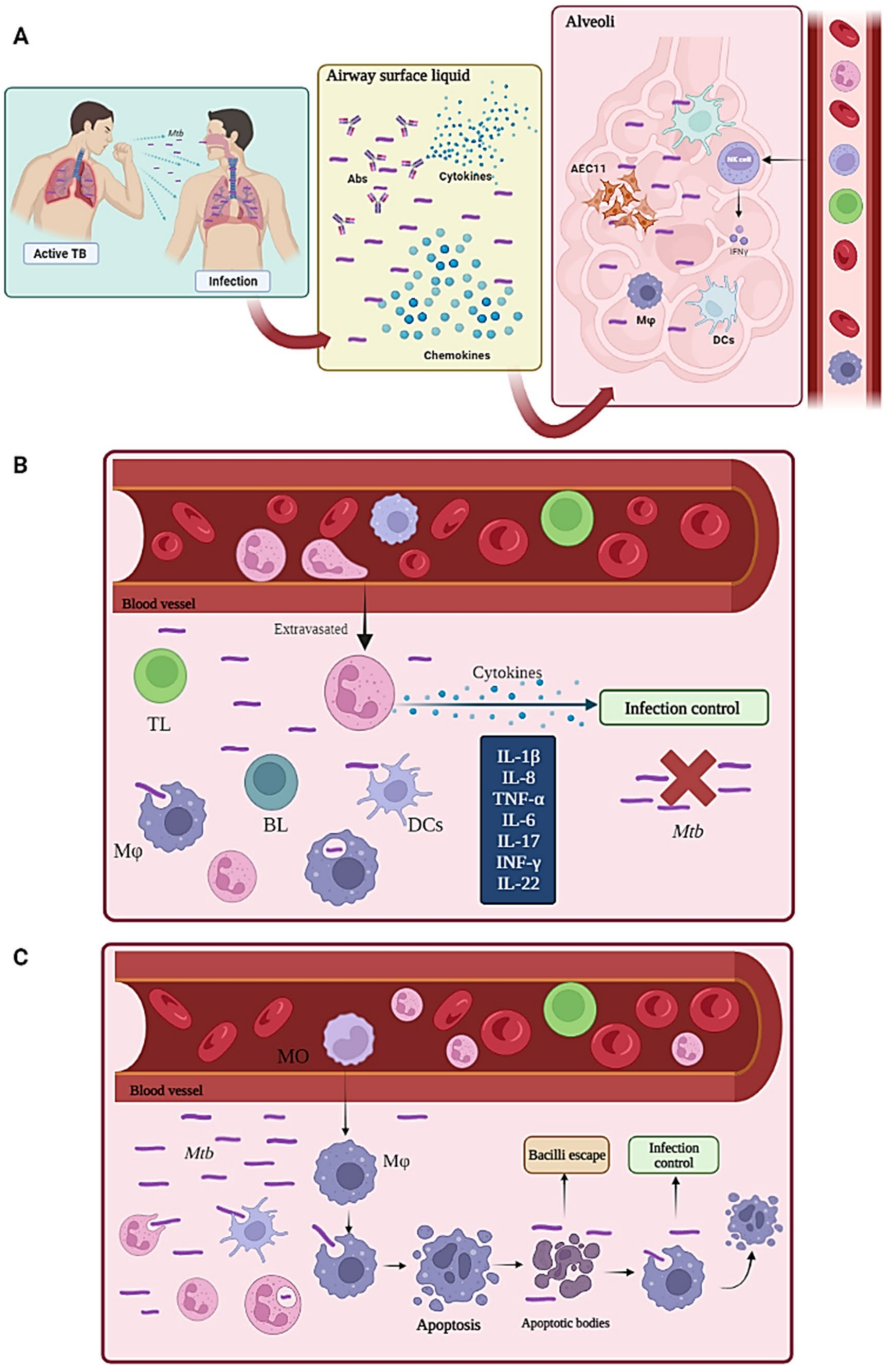
Innate immune response. **A** *Mtb* enters a host when patients having active TB inhale microdroplets which reach the bronchial trees where it then comes into contact with the respiratory mucosa; this is coated by airway surface liquid (ASL), containing antimycobacterial peptides, immunoglobulins, cytokines and chemokines. The microorganism can escape from the respiratory mucosa and reach alveoli made up by type II epithelial cells (AECII), macrophages (MA) and dendritic cells (DCs). Natural killer (NK) cells mediate cell cytotoxicity through IL-2-induced degranulation and cytokine signalling, such as IFN-γ. **B** An innate immune response is mediated by neutrophils (N) which produce cytokines that control *Mtb* infection. **C** Monocytes (MO) differentiate into macrophages (Mφ) which produce apoptotic bodies (apoptosis) promoting the escape of the bacillus, whilst other macrophages phagocytose it and control the infection. Figure created using Biorender.com

Figure 2 shows that airway is the usual way the bacillus enters a host’s organism; the most frequent clinical presentation is thus pulmonary. *Mtb* enters a host when active TB patients’ microdroplets are inhaled (i.e. in which the bacilli are suspended), reaching the bronchial trees where bacteria come into contact with the respiratory mucosa; this is coated with airway surface liquid (ASL) in which there is mucus, antimycobacterial peptides, immunoglobulins, cytokines and chemokines which try to prevent the microorganism’s passage (Fig. 2A).

Some bacilli remain trapped here; however, others manage to pass towards the epithelial cells which have receptors for detecting mycobacteria and induce the release of the elements making up the ASL. Bacilli that have managed to survive the respiratory mucosa meet the lamina propria (LP) containing Mφ and mucosal-associated invariant T-cells (MAIT) which produce IFN-γ, TNF-α and granzyme used for counteracting *Mtb* (Lugton 1999; Middleton et al. 2002; Gold et al. 2010; Harriff et al. 2014).

The microorganism can escape from the respiratory mucosa and reach the alveoli containing type II epithelial cells (AECII) which produce surfactant antimicrobial substances, such as hydrolases, that affect *Mtb* cell wall. The Natural Killer cells (NK) become extravasated; these mediate cell cytotoxicity through degranulation induced by IL-2 and cytokine signalling like IFN-γ (induced by IL-12). It has been shown that IL-12-activated NK cells can inhibit *Mtb* and *Mycobacterium avium* growth and can also develop memory by exposure to antigens, thereby establishing a bridge between innate and adaptive responses (Denis 1994; Bermudez et al. 1995; Garand et al. 2018). It has also been shown that NK cells directly contribute to eliminating *Mtb*-infected cells and increase effective cytotoxic CD8 + lymphocyte function, while some reports have stated that such cells’ function becomes decreased in people having active TB (Vankayalapati and Barnes 2009). NK cells lyse *Mtb*-infected alveolar monocytes and macrophages (AM), produce IL-22 that inhibits mycobacterial intracellular growth and express receptors for soluble factors such as cytokines (Paidipally et al. 2018). NK cells lyse monocytes when healthy donors become infected, thereby reducing *Mtb* intracellular growth (Liu et al. 2017).

Neutrophils are also extravasated to the respiratory mucosa participating in IL-1β, IL-6, IL-8, IL-17, TNF-α, IFN-γ and IL-22 production which are involved in controlling mycobacterial infection. Infection control through neutrophil death by apoptosis has been reported; this induces cross-presentation to antigen-presenting cells (APC), promoting TL-, BL- and NK cell-mediated immunity and Mφ and DC recruitment via chemokines. DCs migrate to lymph nodes for mycobacterial antigen presentation to more lymphocytes; however, polymorphonuclear cells may be unable to clear *Mtb* via phagocytosis, leading to exacerbation of tissue inflammation and damage (Fig. 2B) (Blomgran et al. 2012; Dallenga and Schaible 2016; Warren et al. 2017).

The alveoli also contain resident cells such as alveolar DCs and AM. They control the remaining bacilli through their various defence mechanisms, innate immunity can therefore control *Mtb* and thus purified protein derivative (PPD) or IFN-γ release tests will prove negative. However, it has been well documented that mycobacteria can proliferate due to their immune response evasion mechanisms (as widely reported regarding Mφ) (Chai et al. 2020). APC express a broad diversity of pattern recognition receptors (PRR) on their surface, thereby facilitating bacillus recognition and phagocytosis, i.e. receptors for immunoglobulin fragment crystallizable (Fc) region, TLRs, lectin C-type receptors, complement receptors and scavenger receptors (Kaufmann 2001; Philips and Ernst 2012; Silva Miranda et al. 2012).

Alveolar macrophage (AM) recognition of the bacillus orchestrates monocyte migration in peripheral blood to the infection site and their differentiation into Mφ; Mφ phagocytosed bacilli can die in the phagolysosome.

The bacilli can escape through the apoptotic bodies when apoptosis of infected Mφ is induced and will become phagocytosed by the new Mφ that migrate to the lesion site, thus facilitating a new niche for the microorganisms to proliferate in. It has been described that apoptotic vesicles also facilitate cross-presentation, being phagocytosed by DCs and their HLA-I locus degradation products presented to cytotoxic CD8 + TL. These are significant effectors regarding protective immunity against *Mtb*, given that memory and effector cells are produced which can induce the lysis of infected Mφ, along with contributing to their activation by means of IFN-γ for eliminating mycobacteria (Winau et al. 2006).

### Crossing the bridge towards adaptive immune response

**Fig. 3:**
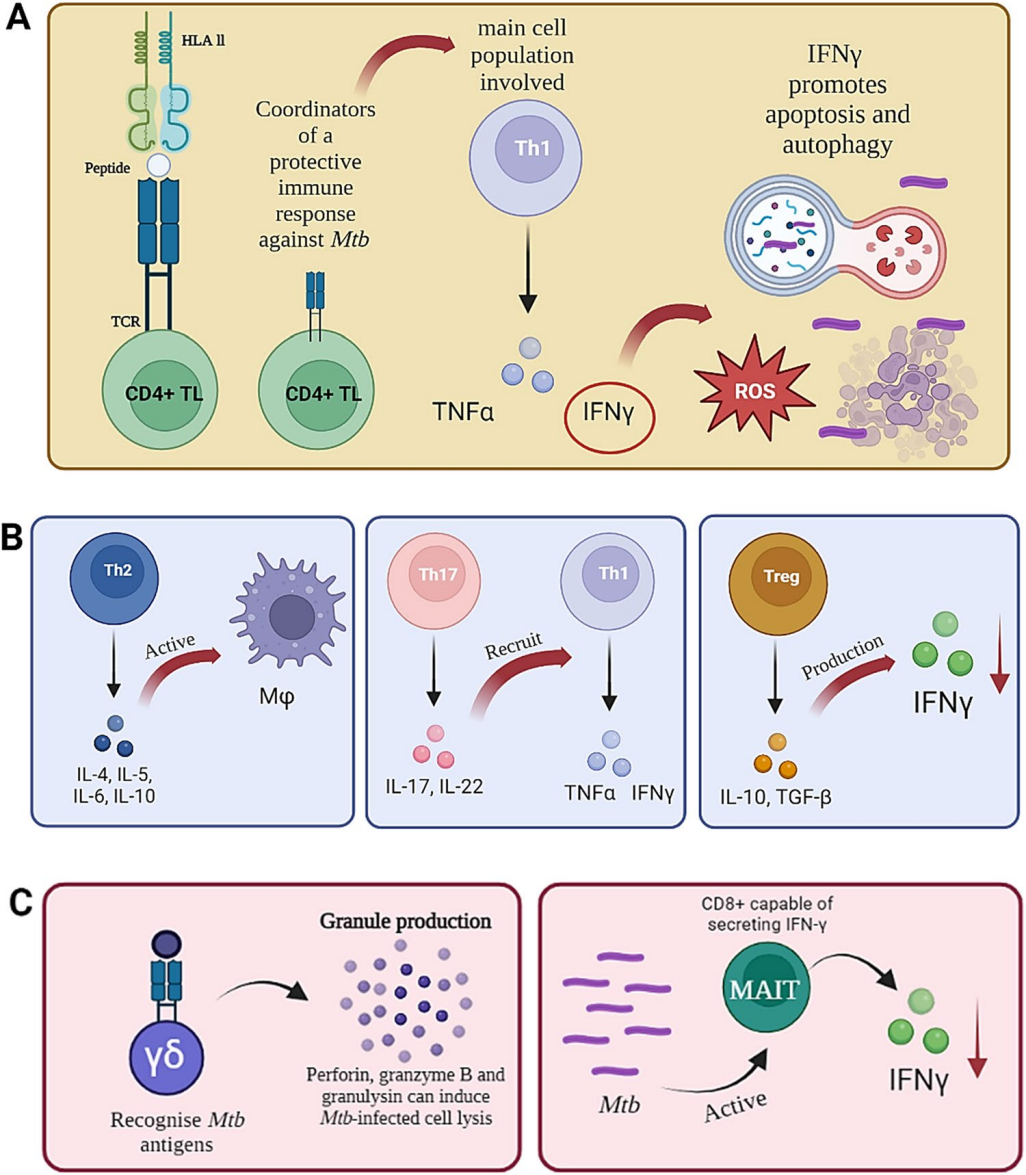
Adaptive immune response. **A** An adaptive immune response is mediated by the HLA-II-peptide-TCR interaction enabling the development of effector memory CD4 + TL. Th1 TL induce reactive oxygen species (ROS)-mediated bactericidal activity, increasing HLA-II expression, promoting apoptosis and autophagy. **B** *Mtb* infection leads to Th2 lymphocytes inducing the production of cytokines associated with Mφ activation, Th17 produces IL-17 (involved in recruiting cells having a Th1 profile) and regulatory T-cells (Treg) which are involved in inhibiting control of *Mtb* infection, decreasing IFN-γ production in people having active TB. **C** Lymphocytes having unconventional receptors, such as gamma/delta (γδ) TCR, recognise *Mtb* antigens and produce significant granules for counteracting *Mtb* infection and MAIT cells that reduce the level of IFN-γ are also present. Figure created using Biorender.com

Figure 3 shows some elements involved in an adaptive immune response, including:

#### CD4+ Th1 TL response

At the same time that mycobacteria are phagocytosed by macrophages, the HLA-II-peptide-TCR interaction enables CD4+ effector memory TL development, together with costimulatory molecules and other signals (Neefjes et al. 2011). Such cells have been described as coordinators of a protective immune response against *Mtb* through a Th1 response. This is an active TNF-α, IL-12 and IFN-γ producer; the latter is considered crucial in a protective response against *Mtb* since it stimulates phagocytic cells to contain intracellular pathogens, promoting different actions, such as bactericidal activity due to reactive oxygen species (ROS), increased HLA-II expression, the promotion of apoptosis and autophagy (Green and Difazio 2013; Chin et al. 2017) and the production of chemokines such as CCL3 for B-lymphocyte recruitment (Saunders and Britton 2007).

#### Follicular helper T-cells

Memory B-cells and Ab-producing plasma cells are produced in the germinal centres (GC), having strong affinity for various antigens. Follicular helper T-cells (Tfh) play an important role in GC formation. These cells express the CXCR5 receptor enabling BL migration to the CG; their formation requires an inducible T-cell COStimulator (ICOS). Tfh also produce IL-21 which (together with CD40L) promotes BL differentiation and isotype switching for producing long-lasting Ab-producing memory and plasma cells (Schmitt et al. 2014). It has been shown that these cells are associated with immune control of tubercle bacilli infection, whilst the absence of CXCR5+ Tfh indicates susceptibility. CXCR5 expression promotes these cells’ localisation in the granulomas, promotes Mφ activation and facilitates lymphoid follicle formation, thus highlighting such cell subpopulation’s importance in the immune response against TB (Slight et al. 2013).

#### Th2 response

Th2 lymphocyte induction for producing IL4, IL5 and IL6 has been described in *Mtb* infection; such cytokines are associated Mφ activation (even though their bactericidal activity against *Mtb* is less efficient) and inhibition of autophagy, associated with mycobacterial intracellular degradation (Harris et al. 2007; Gordon and Martinez 2010). The Th17 lineage produces IL-17 that mediates the recruitment of cells having a Th1 profile; likewise, antimicrobial peptide-inducing cytokines, such as IL-22, IL-26 and granulocyte-monocyte colony-stimulating factor (GM-CSF), stimulate granulopoiesis and granulocyte recruitment and activation (i.e. neutrophils) for eliminating mycobacterial pathogens (Stenger et al. 1998).

#### Regulatory T-cell response

Regulatory T-cells (Treg) are involved in inhibiting *Mtb* infection control since they decrease IFN-γ production in people having active TB, even when faced with potent antigens such as Bacillus Calmette-Guérin (BCG) and ESAT-6, which could contribute to the pathogenesis of the microorganism causing the infection (Chen et al. 2007; Urdahl et al. 2011). They also express granzymes to induce apoptosis in target cells (Grossman et al. 2004), downregulate the expression of costimulatory molecules such as CD80 and CD86 through a CTLA-4-dependent mechanism (Oderup et al. 2006), while they can disrupt Th1 metabolism through IL-2 consumption (Chen et al. 2007).

Tregs are induced by mycobacterial antigens in lymph nodes early on during infection, thus delaying effector TL activation by releasing IL-10 and transforming growth factor β (TGF β) (Kaufmann et al. 2010; Slight et al. 2013). Experiments involving a mouse model have revealed a harmful relationship for the organism with an increase in Tregs, or a beneficial effect to counteract the infection (Ozeki et al. 2010; Cardona et al. 2015). A high level of these cells has been correlated with less pathological damage in non-human primates following IL-2 treatment (Chen et al. 2012) and also high levels in primates which did not develop disease following exposure to the pathogen (Green et al. 2010).

#### Mucosal-associated invariant T-cell (MAIT) response

It has been shown recently that mycobacteria or their antigens can also induce MAIT cell activation and recruitment, as these migrate from the circulation of people having active TB to their respiratory tracts, where they have been characterised as CD8+ capable of secreting IFN-γ, so these cells are an important target for vaccination against *Mtb* (Sakai et al. 2021). MAIT develops in the thymus and presents TCRs from an α-chain paired with β-chains, thus having an important function in the recognition of metabolites presented by MR1-related class I MHC (Sakai et al. 2021).

MAIT TCR recognises a riboflavin-derived, vitamin B-based pyrimidine ligand, presented by MR1 (Xiong et al. 2021). It has been stated that MAIT play a functional role due to their cytotoxic capacity regarding pulmonary epithelial cells expressing MR1 infected by bacteria (Ruibal et al. 2021). Such cells produce cytokines, such as IFN-γ, TNF-α and IL-17, as well as having a direct cytotoxic effect, possibly related to a protector effect against bacterial infections (Jiang et al. 2020).

Transcriptome sequencing (i.e. (RNA-seq)) has been used for studying the functional effect of MAIT cells expressing programmed death receptor 1 (PD-1) in TB patients. It was shown that MAIT cells expressing PD-1 reduced IFN-γ levels; however, CXCL13 and IL-21 levels increased, even though such cells’ presence was more related to an extension of the infection (Jiang et al. 2020).

MAIT cells have also been described as mediators regarding tissue repair. It has been established in some cases that cells reactive to mycobacterial antigens contribute to restricting pathogen growth, although in other cases this has not been directly related, although the lack of these cells’ functionality in mouse model has been linked to increased bacterial load (Gold et al. 2015; Mendy et al. 2018; Pomaznoy et al. 2020; Yu et al. 2020; Vorkas et al. 2020; Ruibal et al. 2021; Xiong et al. 2021; Tukiman and Norazmi 2022). MAIT cell research should thus continue to establish the closest approach to their role in the immune response against *Mtb*.

#### Lymphocytes having unconventional receptors

Other lymphocytes having unconventional receptors have been described concerning the immune response against TB, such as gamma/delta (γδ) TCR. Mycobacterial antigens also come into contact with these lymphocytes (1–5% of circulating T-lymphocytes) triggering cell-mediated immune responses (Dong et al. 2018; Kathamuthu et al. 2021). These cells produce cytokines, such as IFN-γ, TNF-α and IL-17, in response to non-peptide bacterial antigen stimuli, including specific intermediates of the isoprenoid biosynthesis pathway or phosphate analogues (Zufferey et al. 2013).

IFN-γ-producing cells have been observed in BCG immunisation in adults and in children, suggesting that non-conventional lymphocytes play a role in an immune response against *Mtb* which could be used when designing new TB vaccines (Zufferey et al. 2013). It has been stated that these lymphocytes can contribute to inducing effector functions, such as cell proliferation, cytokine release by CD4+ Th1 TL and stimulating CD8+ TL cytotoxic activity (Chen 2013). Cytotoxic granules (granulysins) and other antimicrobial peptides may be produced in *Mycobacterium bovis*-BCG- or mycobacterial lysate-activated cells and cytolytic activity has also been shown after co-cultivation with mycobacterial-infected human monocytes (Chen 2016).

Experiments have shown that γδ T-cells can recognise *Mtb* antigens; such lymphocytes inhibit mycobacteria growth following BCG vaccination, thus conferring protective immunity (Price and Hope 2009). Granule production by cytotoxic T-lymphocytes (CD8+) and γδ TCR has been demonstrated, thereby characterising it as an important mechanism for counteracting infection; proteins such as perforin, granzyme B and granulysin can induce *Mtb*-infected cell lysis, or even directly lyse the bacilli (Stenger 2001). It has been reported that γδ T-lymphocytes promote cytokine and chemokine production and stimulate cytotoxic molecules against TB during early phases of infection, thus contributing to the immune response (Kathamuthu et al. 2021).

#### Non-classical antigen presentation in TB

A CD1-mediated, non-classical antigen presentation mechanism has been observed (i.e. CD1a, CD1b, CD1c and CD1d), in addition to HLA class I and II antigen presentation. Such mechanism involves mycobacterial glycolipids which are abundant in the cell wall, such as phosphatidylinositol mannosides, lipoarabinomannan, mycolic acids and sulfoglycolipids, such presentation usually induces IFN-γ production.

It has been found that CD1a is expressed on cell surface and in early endosomes whilst CD1b is expressed in late endosomes or lysosomes. CD1c and CD1d are located on cell surfaces and are recycled in late endosomes. These molecules are abundant in DCs; however, they are downregulated in mycobacteria-infected cells. CD1d is expressed in haematopoietic and non-haematopoietic cells, including epithelial cells (Kaufmann 2001; Seshadri et al. 2013; James et al. 2018).

CD1b and CD1d molecules have been detected on the surface of Mφ and DC in the granulomas of people having TB; this may indicate these cells’ lipid-specific role during TB that could modulate a positive immune response (Cheng et al. 2017). TNF-α-, IFN-γ- and IL-2-mediated protection against *Mtb*, along with cytotoxic granule expression, have been observed following pathogen challenge when *Mtb*-specific CD1b T-lymphocytes have been transferred (Zhao et al. 2015). The importance of CD1-related immunity against *Mtb* is also supported by reports regarding Vietnamese populations where increased susceptibility to disease was related to functional deficiency due to CD1a polymorphism (Seshadri et al. 2014).

#### Humoral immune response involvement

However, mycobacterial entry to a target host induces both humoral and cellular responses. B-lymphocyte participation in this has been reviewed. Three main facts about these cells have been reported. *Mtb* antigen-specific Ab transfer helps a host control infection (Li et al. 2017), Abs targeting *Mtb* (correlating with lower susceptibility to infection) and TB development is facilitated in hosts having a deficiency regarding Ab or B-lymphocyte production (Casadevall 2004).

BL contributes to an Ab production-mediated humoral response and present antigens which can induce cytokine and chemokine production (Kozakiewicz et al. 2013). These cells’ importance has been clearly reflected in non-human primate studies where BLs have been depleted; it was observed that a granulomatous response could not be modulated during acute TB infection without these cells which attempt to eliminate or contain the mycobacteria to prevent more damaging development of TB (Phuah et al. 2016).

#### TB-associated antibodies

Bacterial attachment to host cell surface is the first step regarding pathogenicity; this enables the pathogen to interact with specific cells, involving different molecules on the pathogen surface (Forthal 2014). Such interaction implies that the microorganism can colonise and invade host tissue; however, they cause damage to host cells since they have several virulence factors (Krachler and Orth 2013). Similarly, *Mtb* entry to host cells is a fundamental event in infection development; inhibition is therefore a key step regarding host protection (Krachler and Orth 2013; Boggiano et al. 2017) so that *Mtb* does not use Mφ and/or other cells as a tool and natural habitat for propagation within a host, thereby causing a more serious pathology (Chen et al. 2012).

Abs acting against *Mtb* have been reported to have direct microbicidal or neutralising activity; they also help enhance phagocytosis to kill the pathogen, increase phagolysosome fusion, restrict *Mtb* growth and promote inflammasome activation in Mφ to kill *Mtb* microorganism (Zimmermann et al. 2016). The amount of mycobacteria became reduced due to Ab action in *in vitro* studies with human cells in whole blood in such a way that various donors’ immunoglobulins were able to control *Mtb* infection (Li et al. 2017). Immunoglobulins acting in protection against *Mtb* infection have affinity for different antigens such as arabinomannan, lipoarabinomannan (LAM), lipoglycoproteins and polysaccharides located on cell envelope; they also recognise proteins such as heparin-binding haemagglutinin (HBHA) and the *Mtb* 16kDA protein (HspX) (Sarmiento et al. 2019).

Zimmermann *et al*., studied IgG and IgA having affinity for mycobacterial antigens in individuals suffering acute pulmonary TB and in healthy subjects exposed to the pathogen. Anti-*Mtb* immunoglobulin A inhibited mycobacterial entry to pulmonary epithelial cells, while IgG (commonly produced during inflammatory processes) had the opposite effect, promoting infection (Zimmermann et al. 2016). It has been reported that people having latent and active TB produce Abs having different effector functions; 70 characteristics of the immunoglobulin Fc from both groups of donors were profiled. It was found that Abs from individuals having latent TB had better activity through FcγRIII (CD16) receptors; they are involved in Ab-dependent cellular cytotoxicity. These immunoglobulins have different glycosylation patterns which lead to the intracellular killing of mycobacteria in human macrophages, thereby promoting enhanced phagolysosomal maturation, inflammasome activation and decreased mycobacterial burden, compared to the action of Abs from people having TBA (Lu et al. 2016). The same group later established that people having latent TB and those having active TB tuberculosis had differential patterns regarding Fc glycosylation of their Abs, directly influencing Ab ability to form part of a protective immune response against mycobacteria (Lu et al. 2020).

The passive transfer of sera recognising pathogen antigens is another research tool regarding the role of the humoral immune response; it is suggested that the Abs contained in them become protective against TB (Hamasur et al. 2004; Buccheri et al. 2009; Balu et al. 2011; Li et al. 2017). It has been shown that Ab targeting arabinomannan and LAM have induced mycobacterial phagocytosis by Mφ in sera from BCG vaccinated people and improved phagolysosome fusion, thereby inhibiting the pathogen’s intracellular growth (Chen et al. 2016). It has been observed that Abs from people having latent TB and healthy subjects have restricted mycobacterial growth more than Abs from people having active TB (Lu et al. 2016; Pai et al. 2016; Li et al. 2017; Carabali-Isajar et al. 2020).

In different stages of *Mtb* infection (latent TB and active TB), it was established via intracellular growth inhibition assays in Mφ cell lines that the IgG anti-AM (arabinomannan) produced naturally during asymptomatic individuals’ (TBL) infection was protective, but not in sick donors (TBA) (Chen et al. 2020). Fischinger *et al*., evaluated a cohort of HIV-infected people previously treated for TB with and without recurrent disease, for establishing whether there are protective Abs amongst individuals who do not have recurrent TB and individuals who do. Given that TB is the main cause of death in HIV-positive people, it was found that those infected with recurrent TB had lower *Mtb*-specific IgG3 titres; these were preserved in control individuals who did not suffer frequent infection, while the amounts of the other IgG or IgA subclasses were equal in both groups (Fischinger et al. 2021).

Rhesus macaque monkeys (*Macaca mulatta*) intravenously immunised with BCG had IgM Ab titres which correlated significantly with a decrease in *Mtb* load in the lungs. *Mtb*-specific IgM monoclonal Ab (mAb) were obtained that reduced pathogen *in vitro* survival (Irvine et al. 2021). Protection was observed against challenge with *Mtb* using this same animal model in another report; this was correlated with increased IgA induced by BCG vaccination using the bronchial instillation technique (Dijkman et al. 2019).

The MTBVAC HK vaccine has been used as a booster in macaques after BCG. Immunoglobulins that induced mycobacterial opsonisation *in vitro* was evidenced which was associated with increased human Mφ capability to restrict the bacteria in acidic intracellular compartments (Aguilo et al. 2020). The ability of human sera recognising *Mtb* surface protein-derived peptides to inhibit pathogen entry to human Mφ was determined *in vitro* later on; it was verified that these Abs had the same inhibitory function after isolating peptide-specific IgG Abs. Non-human primates from the genus *Aotus* were subsequently inoculated with peptides to evaluate the activity of the Abs so produced; it was observed that these animals’ sera also reduced the percentage of pathogen entry to human Mφ (Carabali-Isajar et al. 2020).

**Fig. 4:**
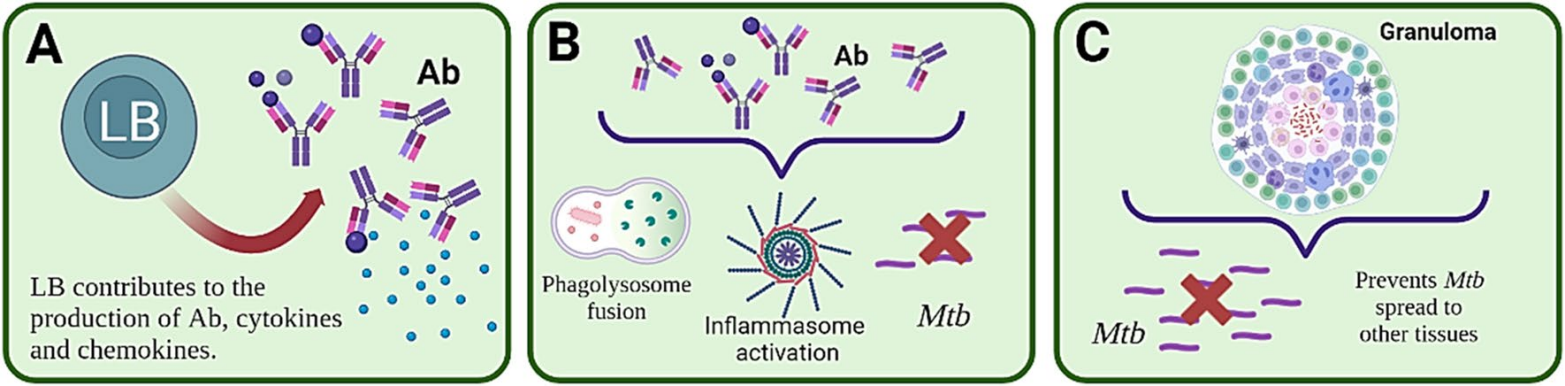
Humoral immune response. **A** BL contribute to an Ab production-mediated humoral response and present antigens that can induce cytokine and chemokine production. **B** Abs acting against *Mtb* have been reported to enhance phagocytosis for killing the pathogen, increase phagolysosome fusion, restrict *Mtb* growth and promote inflammasome activation in Mφ to kill *Mtb*. **C.** Granuloma prevent *Mtb* spread to other tissues and lead to its rapid destruction within such granuloma

Some authors have reviewed B-lymphocytes’ role in GC which is fundamental for the development of Ab-producing plasma cells, in addition to the importance of producing memory cells that also contribute to preventing TB development (Lyashchenko et al. 2020; Rijnink et al. 2021). The foregoing observations show that Abs and BL have many mechanisms enabling a host to cope with *Mtb* as seen in Figure 4.

#### Granuloma

Inflammatory cytokine and inflammatory chemokine production occurs in the population that has not had previous exposure to the mycobacteria once the PRR expressed in lung cells (i.e. Mφ, DCs and epithelial cells) interact with mycobacterial ligands. Such inflammatory elements are responsible for recruiting new cells at the primary infection site and also trigger granuloma formation by the innate immune system. The adaptive immune response usually occurs 4 to 6 weeks after DC antigen presentation in regional lymph nodes (Fig. 4).

The adaptive immune system has a predominance of delayed Th1:Th17-type response in TB and can offer protection against infection by containing the mycobacterium in a granuloma; these are cellular aggregates, formed by: alveolar macrophages, pneumocytes, DCs, neutrophils, macrophages derived from monocytes, occasionally it is find multinucleated giant cell, epithelioid macrophages, NK cells, αβ and γδ T-cells, fibroblasts, BL, plasma cells and also the presence of Abs has even been described (Sholeye et al. 2022; Ashenafi and Brighenti 2022; Ehlers and Schaible 2013; Kozakiewicz et al. 2013). The granuloma prevents *Mtb* spread to other tissues and leads to its rapid destruction within such granuloma that reaches structural and functional maturity with the adaptive immune response. Innate immune system infection control is not efficient in some cases; it has thus been suggested that this is why mycobacterial spread to other tissues is enabled (Cadena et al. 2016; Ankrah et al. 2018).

#### Complement systems as part of host defence against the pathogen

It is known that the complement mechanism also acts against mycobacterial infections since the membrane attack complex (MAC) can be created through the classical pathway (involving Ab action) through which mycobacterial lysis is induced (Manivannan et al. 2012; Kouser et al. 2013; Forthal 2014). Complement system elements can contribute to mycobacterial entry to host cells since phagocytes have several complement receptors. Mφ complement receptors can be manipulated by mycobacteria and used as a reservoir for it (Ferguson et al. 2004; Carroll et al. 2009; Jagatia and Tsolaki 2021). It is known that this system also produces molecules known to be proinflammatory such as C3a, C5a (Coulthard and Woodruff 2015; Wu et al. 2022; Ito et al. 2022) and factor H from the complement positively regulating TNF-α, IL-1β and IL-6 production (Kouser et al. 2013; Abdul-Aziz et al. 2016).

### Trained immunity’s contribution to defence against TB

Host immune system mounts a response after infection by or exposure to an antigen; this is known as trained immunity which induces a more robust response when an organism is exposed to an agent similar to or even different from the one that produced the first immune reaction (Netea et al. 2020). It is known that type I and type II interferons secrete certain BCG-induced elements in TB-related infections; β-glucans also induce proinflammatory cytokines, such as IL-1 and granulocyte colony-stimulating factor (G-CSF) and Mφ (Kaufmann et al. 2018; Mitroulis et al. 2018; Moorlag et al. 2020; Khan et al. 2020).

Joosten et al*.,* used a mycobacterial growth inhibition assay in which BCG growth was seen to be controlled by sera from donors recently exposed to *Mtb*; however, such response was much lower in people having latent TB and those vaccinated with BCG. Trained immunity effector mechanisms were related to TL and monocytes (Joosten et al. 2018). Considering that BCG can induce trained immunity, the mechanisms that may be involved in it have been investigated; BCG can induce the epigenetic and metabolic reprogramming of cells such as Mφ, which allows a non-specific response to occur upon stimulation, but induces greater production of proinflammatory cytokines and microbicidal substances, such as ROS. Moreover, BCG vaccination in healthy humans has been seen to induce changes in neutrophil phenotype which are associated with epigenetic modifications of the genome, a greater expression of activation markers having an antimicrobial function being evidenced (Verrall et al. 2014; Crișan et al. 2016).

Lipopolysaccharide (LPS) represent another mechanism capable of inducting trained immunity; like BCG it induces different gene expression patterns resulting in the elimination of invading pathogens (Verrall et al. 2014; Morris et al. 2014; Crișan et al. 2016).

It has been described that LPS can activate Mφ through TLR4; this is related to a greater capacity for phagocytosis and ROS production. This results in *Mtb* elimination, thereby promoting a protective immune response (Jo et al. 2007; Lv et al. 2017). This may be linked to why it is quickly controlled, despite being exposed to *Mtb* during the early stages of infection; this prevents disease development without leaving an immunological trace of the pathogen in human immune system, as shown by studies in populations which have been widely exposed to mycobacteria (Behr et al. 2019; Verrall et al. 2020; Chihota et al. 2022).

### Immune system evasion: how *Mycobacterium tuberculosis* manages to survive host immune system response

As previously noted, a host is armed with different branches of immunity to prevent TB development; however, each year there are reports of different mechanisms through which mycobacteria escape host immune system control (Fig. 1). The most known evasion mechanisms are thus summarised in the following section where it can be seen that the mycobacteria induce responses in a host enabling their survival and multiplication.

#### Action on Mφ and DCs

Mycobacteria manipulate these cells’ receptors, thereby decreasing their activation (Zhu et al. 2014; Dabla et al. 2022), delaying DC migration to lymph nodes to affect T-lymphocytes effector production (Boggiano et al. 2017). They induce Mφ recruitment whose growth is tolerated via chemokine CCL2 which is produced by microorganism-infected cells (Ernst 2012). Mycobacteria use alveolar Mφ to evade detection and the activation of cells capable of eliminating them; while bacterial dissemination begins in the lung interstitium through these cells (Queval et al. 2017; Cohen et al. 2018).

Mφ can become a niche for the pathogen when recruited Mφ offers no microbicidal response and they have become infected by *Mtb*; the mycobacteria thus gradually replicate (Queval et al. 2017; de Martino et al. 2019).

#### Antigen processing and presentation

Mycobacteria inhibit MHC-II synthesis which leads to reduced CD4 + TL-mediated immunity action due to a lack of antigen presentation (Noss et al. 2001; Dolasia et al. 2021). *Mtb* can avoid and/or tolerate phagolysosome formation; this directly affects phagocyte maturation, limits lysosome formation and inhibits phagosome acidification (Wong et al. 2013; Chen et al. 2015; Sajid et al. 2015).

The PKnG protein helps enhance the pathogen’s growth rate, virulence, drug resistance and prevents phagosome and lysosome fusion (Walburger et al. 2004; Wong et al. 2013; Sajid et al. 2015). The pathogen is released from the phagosome into the cytosol, evades phagolysosomal fusion and continues its growth and survival (Dallenga et al. 2017; Kroon et al. 2018). *Mtb* is also able to suppress phagosome acidification by maintaining a ~ 6.2 pH, at which mycobacteria can survive (Chen et al. 2015).

#### TL-mediated immune response

The bacteria prevent and delay CD4 + and CD8 + T-cell response activation when *Mtb* infection occurs; this decreases mycobacterial antigen specificity and therefore makes the pathogen’s efficient detection of infected cells more difficult (Yang et al. 2018; Patankar et al. 2020).

Mycobacteria can directly affect T-cells, thereby decreasing the phosphorylation of factors which can block TCR signalling and thus prevent correct lymphocyte activation (Mahon et al. 2012) and induce antigen-specific Treg lymphocytes that delay T-lymphocyte effector presentation and recruitment to the granuloma (Ramakrishnan 2012). The pathogen promotes the synthesis of suppressor factors restricting T-lymphocyte effector function, thereby preventing their immune response from focusing on conserved and/or subdominant regions (Goldberg et al. 2014).

#### Regulating granulomas

*Mtb* activates genes helping it survive in conditions limiting growth, such as hypoxia and lack of nutrients (Kroon et al. 2018); however, although granulomas prevent bacterial spread to extrapulmonary sites, they can also become mycobacterial reservoirs from which mycobacteria can escape, thereby leading to the active form of the disease (Dallenga et al. 2017).

#### Human polymorphonuclear neutrophils (PMN)

Neutrophils (PMN) are haphazardly recruited to an infection site, thereby exacerbating inflammation and thus increasing disease severity (Kroon et al. 2018). The mycobacteria lead infected neutrophils to necrosis; this promotes pathogen growth as more Mφ and PMNs are recruited, in turn becoming rei-infected and thus prolonging the mycobacterial life-cycle (Repasy et al. 2015; Dallenga et al. 2017).

#### Virulence factors and survival

PE/PGRS proteins may play a role in promoting mycobacterial survival by modulating proinflammatory cytokines which interfere in lipid metabolism when a host interacts with Mφ proteins, thereby leading to its malfunction. Mφ arachidonic acid metabolism also becomes altered which also affects microbicidal capacity (Ramakrishnan 2012; Goldberg et al. 2014) by masking pathogen-associated molecular patterns (PAMPS) with lipids from the cell surface to avoid recruitment of Mφ microbicides which can eliminate it (Barberis et al. 2017).

### Clinical manifestations of TB: what happens if mycobacteria evade human immune system checkpoints and manage to colonise different host organs?

Understanding TB’s clinical spectrum involves understanding that this is a disease that has evolved along with our species. The first *Mtb* variant originated in East Africa about 3 million years ago (Barberis et al. 2017) and the strains circulating today originated from a common ancestor around 20,000–15,000 years ago. Egyptian and pre-Columbian mummies carry sequelae of Pott’s disease (or spinal tuberculosis), or *Mtb* DNA has been isolated from them (Donoghue et al. 2010).

Such long-running association has produced a complex series of adaptations and counteradaptations between a host and the mycobacteria. As discussed above, mycobacterial transmission occurs via airborne particles (Flügge droplets) containing one to five *Mtb* bacilli (Murillo-Godínez 2009). They reach the alveoli after having been inhaled, being phagocytosed by alveolar Mφ, neutrophils and DCs, inside which the mycobacteria replicate freely as they are protected from lysosomal enzyme action by their complex wall, medium alkalinisation and the inhibition of various lysosomal pathways (Chandra et al. 2022).

Mycobacteria induce Mφ necrosis and the recruitment of more Mφ and myeloid cells, thereby enabling further replication to occur (Chandra et al. 2022). Antigen-presenting cells then activate CD4 + TL, initiating a type 4 hypersensitivity reaction (Sia and Rengarajan 2019). This first contact is known as primary infection, or primary TB, and can culminate in the development of a hypersensitivity reaction (Bentabol Moreno 1983); this can occur at any age, and several outcomes can occur from this point onwards. Complete cure may occur and the disease may not develop; infection may become controlled by viable bacilli in a latent form that may or may not induce disease development in future life. Progressive disease may develop, a fact that is more frequent during the first 5 years of the infection (Yew et al. 2018; Alzayer and Nasser 2022).

The vast majority of TB-infected people will be asymptomatic after primary infection, either because the disease has become controlled or because the mycobacterium remains in a latent state, but able to develop into active disease in the future. It has been estimated that between 5 and 15% of people having latent TB will develop the active disease (Shah and Dorman 2021). Some groups of people are at an increased risk of developing active TB from the disease’s latent form; the definition of these groups varies in relation to local epidemiology, usually being HIV-infected people or recent contacts of a person having active TB or people having fibrotic changes in chest x-rays consistent with old TB. They may also have a positive interferon-gamma release assay (IGRA) or tuberculin skin test (TST) reaction of 5 or more millimetres at high risk of developing active disease and should receive anti-tuberculosis treatment to reduce such risk (Centers for Disease Control and Prevention 2022).

The term progressive TB or reactivation denotes TB progression; progressive TB is uncontrolled primary infection that progresses to active disease whilst reactivation is latent TB that is activated and progresses. Although there are pathophysiological differences from a clinical point of view, they cannot be differentiated (Moule and Cirillo 2020). Active TB can cause lung disease and/or spread from the lungs to other organs by haematogenous, bronchogenic and lymphatic routes. Such dissemination gives rise to clinical pulmonary TB and extrapulmonary TB presentation (Moule and Cirillo 2020).

### Pulmonary tuberculosis

The lungs are the most commonly affected organ in an immunocompetent host during TB infection. Lung involvement in subjects having active TB has been estimated at 79–87%, whilst the estimate of lung involvement is similar in immunocompromised hosts, like those having HIV–HIV infection (70–92% lung involvement rate). Symptoms are constitutional and pulmonary, their frequency depending on whether the patient has primary TB or reactivated TB. Subjects having primary TB are much more likely to be asymptomatic or minimally symptomatic (Lyon and Rossman 2017).

Pulmonary TB is usually a disease having an insidious onset. Fever is the most frequently observed constitutional symptom which characteristically develops in the late afternoon. There may be other symptoms in up to 75% of cases of pulmonary TB, such as malaise, weakness, unusual fatigue, headache, night sweats and weight loss. This is usually accompanied by caseous necrosis and concomitant caseous liquefaction and cough and purulent sputum which is often associated with mild haemoptysis. Chest pain can be pleuritic and localised. Dyspnoea and respiratory distress usually indicate extensive disease, along with widespread involvement of the lungs and parenchyma or some form of tracheobronchial obstruction, therefore usually occurring late in the course of TB (Heemskerk et al. 2015; Lyon and Rossman 2017). Lung involvement can be cavitary, bronchial or miliary, depending on immune status or the type of dissemination (Heemskerk et al. 2015).

Cavitary involvement is perhaps the best-known pattern of TB lung injury worldwide. Cavitation formation is a complex process involving a series of biochemical and immunological processes. *Mtb* does not have toxins or lytic enzymes; tissue is thus destroyed by the immune response itself. Cavitation formation occurs when granulomas become formed via the type four hypersensitivity reaction, leading to pathological remodelling of the matrix. Alveolar tissue becomes effaced and is replaced by fibrotic matrix deposited by fibroblasts. Loss of basement membrane causes irreversible damage to the lungs and is probably due to immune cells moving into the centre of a granuloma, secreting matrix-remodelling proteases to aid migration. Caseous debris formed by caseation necrosis in the centre of the area of granulomatous pneumonia is evacuated through the bronchial tree (Urbanowski et al. 2020).

Such remains are particularly smear-positive, making the cavitary form of TB one of the main sources of contagion for other people (Caminero 2006; Urbanowski et al. 2020). Diagnosis is made from radiological findings and microbiological confirmation of the microorganism by Ziehl–Neelsen staining, culture or molecular tests. Treatment must be prolonged to guarantee cure due to high bacillary load and the difficulty of anti-tuberculous drugs to reach therapeutic concentrations in cavitations.

Bronchial involvement is also called bronchogenic or bronchogenic TB since it spreads through the bronchi instead of the lymphatic vessels or the bloodstream. This begins as a prolonged asymptomatic accumulation of mycobacterial antigens and host lipids in the alveolar Mφ. Clinically, it is characterised by cough and expectoration, although having lower bacillary load than in cavitary disease. Granulomas in bronchogenic TB are morphologically different from those in cavitary TB (Hunter et al. 2018). Radiological characterisation involves pulmonary nodules in the centre of the secondary pulmonary lobule, having ramifications known as a budding tree pattern (Hansell et al. 2008).

Miliary TB involves bloodborne spread of TB. The pulmonary manifestation of haematogenous dissemination of TB is characterised by the appearance of multiple, smaller than 1 mm nodules diffusely distributed throughout lung parenchyma. The term miliary TB was coined around 1700 by John Jacob Manget, alluding to the similarity between the lesions found in this type of TB and millet seeds (Sharma and Mohan 2017). This type of TB used to occur more frequently in children; however, the use of the BCG vaccine has decreased its frequency in this age group (Zhu et al. 2018). Other factors explain miliary TB’s changing epidemiology, such as the AIDS pandemic, the availability and widespread use of computed tomography and the increasing frequency of solid organ transplants (Sharma and Mohan 2017; Giacomelli et al. 2018).

Cases of miliary TB from pulmonary TB have been described as a paradoxical reaction to anti-tuberculosis drugs (Izquierdo et al. 2020). Although pulmonary involvement is the most obvious cause, haematogenous spread enables mycobacteria to reach almost any organ in the body. Miliary lesions can be found in the lung, liver, spleen, lymph nodes, meninges, bone marrow and adrenal glands (Sharma and Mohan 2017). Clinical presentation is variable, the clinical picture in miliary TB having a systemic component including weight loss and nocturnal diaphoresis and an organ-specific one that includes haematological, renal and neurological involvement. Severity can be extremely variable, ranging from insidious forms having non-specific symptoms to acute severe forms involving septic shock, multiple organ failure and acute respiratory distress syndrome (ARDS). Miliary TB is a known, though rare, cause of ARDS (Sharma and Mohan 2017).

### Extrapulmonary tuberculosis

Pulmonary disease occurs in more than 80% of TB cases; however, any region of the body can become affected during mycobacterial infection which can use bronchogenic, lymphatic or haematogenous pathways to spread to other neighbouring or distant organs in the body from an initial source of infection. Extrapulmonary TB occurs in around 20% of cases, but it is more frequent in the immunosuppressed population (as in HIV), reaching more than 50% of cases. Active TB presentation can be highly variable, ranging from a state in which there are no symptoms to severe associated sequelae, such as Pott’s disease, or even become life-threatening, as in the case of tuberculous meningitis (Sharma and Mohan 2004; Gambhir et al. 2017).

TB bacilli affect pulmonary and mediastinal hilar lymph nodes whilst undergoing haematogenous and lymphatic dissemination during primary infection; this leads to primary Ghon complex formation constituted by tuberculous lung parenchyma lesions (single and peripheral) and pulmonary hilum lymph nodes so involved to which the affected area drains. Bacilli entering the bloodstream or lymphatic system spread, resulting in extrapulmonary TB during primary infection or later on during reactivation of the disease. The lymph nodes are the most commonly affected site by extrapulmonary TB; however, pleural, neurological, synovial, musculoskeletal, pericardial, peritoneal and genitourinary involvement have also been described (Natarajan et al. 2020).

Some risk factors promote the spread of mycobacteria outside the lungs, such as age, being female, HIV infection and comorbidities, such as chronic renal failure, diabetes mellitus or immunosuppression (Ramírez-Lapausa et al. 2015). Extrapulmonary TB is a major diagnostic challenge since sputum analysis is not available for diagnosis and sampling is often required for invasive methods to confirm diagnosis (Moule and Cirillo 2020).

#### Tuberculous lymphadenitis

Tuberculous lymphadenitis was called scrofula and considered a different disease to TB until Robert Koch (1882) was able to isolate the *Mtb* bacillus from the ganglia of 33 affected people, demonstrating that it was indeed TB-related extrapulmonary compromise (Duarte 2017). This clinical picture constitutes about 35% of extrapulmonary TB cases and the most commonly affected body area is the cervical region with 60-90% of reported cases. Cervical lymph node involvement is due to lymphatic dissemination of bacilli from the Ghon complex or from the tonsils, adenoids, or from the involved ethmoid bone. Once mycobacterial colonization occurs, the bacilli proliferate inside the lymph nodes, generating local inflammatory-inflammatory changes such as marked hyperaemia, oedema, necrosis, and caseation of the compromised lymph node.

Such clinical picture constitutes about 35% of extrapulmonary TB cases; the most commonly affected body area is the cervical region, accounting for 60–90% of reported cases. Cervical lymph node involvement is due to lymphatic dissemination of bacilli from the Ghon complex or from the tonsils, adenoids or from involved ethmoid bone. Once mycobacterial colonisation occurs, the bacilli proliferate inside the lymph nodes causing local inflammatory changes, such as marked hyperaemia, oedema, necrosis and caseation of compromised lymph nodes.

Inflammatory changes spread regionally in the area of the involved ganglion chain following the spread of bacilli to adjacent nodes causing adjacent skin to adhere or nodes to rupture into surrounding tissues or through the skin, forming sinuses or fistulous tracts. Compromise of the mediastinal ganglion chains can cause compression of structures, such as major blood vessels and the recurrent phrenic or laryngeal nerves, or even cause bronchial. The detailed mechanisms of bacterial spread remain unclear. Bacilli must traverse the alveolar epithelium to reach draining lymph nodes and the bloodstream. It has been shown that mycobacteria within alveolar Mφ or DCs can be relocated to lymph nodes and blood and that mycobacteria can also invade and lyse epithelial cells after infecting them (Cataño and Robledo 2016).

Some experimental studies have explored the cellular and molecular immunological mechanisms enabling mycobacterial colonisation in the lymph nodes, along with associated inflammatory processes. Once the Mφ and DCs phagocytose mycobacteria in the lungs, they transfer them to the lymph nodes where they become localised through CCR7 receptor interaction with the CCL19/21 ligand expressed in lymph nodes’ endothelial cells. DCs produce a sufficient amount of IL-12p40, IL-12Rβ1 and CD11c here and initiate the immune response by Th1-cell priming, leading to memory effector cell production and such inflammatory response promotes specific granuloma formation.

Mycobacteria can be lysed in phagolysosomes, killed in apoptotic Mφ or killed by NK or CD8+ cells. Adequate amounts of IL-12p70 in the lymph nodes ensures sufficient Th1 cell and IFN-γ production. IL-23 and IL-27 are released during early stages of mycobacterial infection in the lungs and the lymph nodes. IL-23 potentiates IL-17 production and IL-27 promotes IFN-γ production (Averbakh and Ergeshow 2018). A classic interpretation of tuberculous lymphadenopathy concerns an epithelioid granulomatous reaction occurring, accompanied (or not) by caseation and necrosis; some nonspecific lymphoid infiltrates, noncaseating granulomas or Langhans giant cells can be found (Cataño and Robledo 2016). TNF-α is a multipotent cytokine that plays roles in apoptosis, activation, differentiation and cell recruitment in inflammatory-inflammatory foci.

Regarding TB, TNF-α is involved in the differentiation of T-cells secreting Th1 cytokines, granuloma formation with phagocytic Mφ and epithelioid cell activation and mycobacterial death, together with IFN-γ, stimulation of apoptosis of mycobacteria-containing Mφ, stimulation of chemokine production (CCL-2, -3, -4, -5, -8) and endothelial cell adhesion molecule expression (CD54), leading to cell accumulation in inflammatory foci. Type II interferons (IFN-γ) have been the most studied cytokines regarding anti-tuberculous immunity due to their great importance for phagocytosis and subsequent mycobacterial death. IFN-γ is mainly produced by activated CD4+ and CD8+ T-cells and, to a lesser extent, by γδ T-cells, NK T-cells and innate immunity-related NK cells.

The strongest IFN-γ gene expression is detected in activated Th1-cells stimulating Mφ to eliminate mycobacteria, enhance other cells’ cytotoxic activity, induce apoptosis in skin and mucosa epithelial cells, regulate MHC class I and II protein expression and antigen presentation. IL-10 is an inflammatory response multifunctional regulatory cytokine and has been shown to act as a general inhibitor of Th1 and Th2 cell proliferative responses. IL-10 regulates inflammation by suppressing the production of cytokines such as IL-1α, IL-1β, IL-6, IL-8, IL-12 and TNF-α in activated Mφ and IFN-γ in T-cells. TGF-β is the main representative of the family which currently consists of 35 factors, including 5 TGF-β isoforms, bone morphogenic protein (BMP), growth differentiation factors (GDF) and activin and inhibin factors. TGF-β is secreted in an inactive form (L-TGF-β); it is activated after the action of plasmin, thrombospondin-1, reactive oxygen radicals and αVβ6 integrin.

Concerning TB, antigen-regulatory antigen-specific Th1 is the main IL-10 producer and Th3 the main TGF-β one; they are activated upon cooperation with DCs and produce optimal interaction balance with specific effector T-cells and control tuberculosis infection regarding an excessive immunopathological response (Averbakh and Ergeshow 2018).

#### Pleural tuberculosis

TB-related pleural involvement is the second most frequent after nodal involvement; its incidence can be 20–30% of all extrapulmonary TB cases. Pleural TB is paucibacillary TB, meaning that there is a poor bacillary load. The pleural space becomes infected from initial lesions of the lung parenchyma, leading to an immune response with a predominance of neutrophils during the first 24 hours, followed by Mφ reaching their maximum point of action after 96 hours. A lymphocyte-mediated immune response finally occurs, with consequent pleural granuloma formation and adenosine deaminase (ADA) release (Ramírez-Lapausa et al. 2015; Shaw et al. 2018).

Pleural effusions were previously thought to be a purely Th1-mediated delayed hypersensitivity reaction due to mycobacterial antigen entry to the pleural space from ruptured subpleural caseous foci. However, recent evidence has shown that they can also be paucibacillary infections, most likely spreading directly from associated parenchymal lesions. Several findings have supported a delayed hypersensitivity response as a pleural effusion mechanism. An exudate-type effusion developed after introducing mycobacterial antigens into the pleural space of PPD sensitised guinea pigs, effusion became suppressed after antilymphocyte serum administration in the same guinea pig model, historically low mycobacterial culture yield in pleural fluid lymphocyte predominance in most aspirated fluids, with a high percentage of T-cells compared to serum/peripheral blood, and high IFN-γ- and Th1-related cytokine levels (e.g. IL-12) in pleural fluid (Vorster et al. 2015; Koh 2017).

Findings supporting direct pleural infection as a pleural effusion mechanism include microbiological evidence of pulmonary disease in many cases, up to 76% positivity rate of pleural effusion culture for mycobacteria evidence that a significant percentage of tuberculous effusions are neutrophilic (at least at an early stage), identifying a negative association between the percentage of lymphocytes in pleural fluid and the probability of a positive effusion culture result (suggesting infection and clearance) and higher mycobacterial load in loculated effusions. The initial response to mycobacteria in the pleura has been shown to consist of a rapid influx of polymorphonuclear leukocytes, particularly neutrophils which remain in predominant cells for the first 24 hours, followed by Mφ, which peak after 96 hours, and lymphocytes (Vorster et al. 2015; Koh 2017; Shaw et al. 2018).

A recent theory has postulated a continuum spectrum of tuberculous pleurisy, suggesting that TB empyema are predominant in lymphocytes during initial testing. However, the fluid becomes predominantly neutrophilic as the disease progresses, along with the development of loculations and positive spill cultures. Fluid accumulation in the pleural space increases capillary permeability due to the inflammatory reaction with the subsequent influx of proteins stimulating a higher pleural fluid formation rate. Fluid drains through openings in the parietal pleura called pleural lymphatic stomata; however, diffuse parietal pleura involvement by mycobacteria and damage to or obstruction of the stomata leads to a decreased fluid removal rate and subsequent pleural effusion (Vorster et al. 2015; Koh 2017; Shaw et al. 2018).

Typical clinical manifestations usually consist of an acute febrile syndrome, non-productive cough, pleuritic chest pain, dyspnoea, night sweats, chills and weight loss. Diagnosis is based on isolating the germ in pleural fluid (which is rare due to low bacillary load), determining granulomas in biopsy of the pleura or ADA levels being determined in a suitable context (Valdés et al. 1996; Zhai et al. 2016). Tuberculous empyema is an unusual presentation of TB in the pleura, it involves the active mycobacterial invasion of the pleural cavity, usually due to the rupture of a cavitation near the pleural cavity. The resulting inflammation results in pleural effusion having a predominance of neutrophils in its cellularity and abundant *Mtb* in the pleura. Unlike pleural TB, tuberculous empyema requires surgical drainage for management (Zhai et al. 2016). When chronic tuberculous empyema becomes resolved it leaves a thickened, scarred and calcified pleura causing chronic chest pain, dyspnoea and impaired lung function (Koh 2017).

#### Tuberculosis of the central nervous system

Central nervous system (CNS) disease caused by mycobacteria is among the least common, but most lethal forms of *Mtb* infection. *Mycobacterium* that has spread from another focus reaches the CNS and crosses the blood-brain barrier via infected monocytes/neutrophils and can cause caseating foci (Rich foci). Such foci can break into the subarachnoid space, triggering a T-cell response having high IFN-γ and TNF-α levels in cerebrospinal fluid (Leonard 2017).

CNS infection comprises a spectrum covering subacute or chronic meningitis, intracranial tuberculoma and tuberculous spinal arachnoiditis. All three forms can be observed in regions of high TB prevalence where there is clinical post-primary extrapulmonary infection among children and young adults. Tuberculous meningitis initially involves haematogenous mycobacterial dissemination from a primary pulmonary focus or late reactivation in another body region. The blood-brain barrier is not breached in such mycobacteremia to produce immediate direct invasion of the meninges and subarachnoid space, but rather a scant number of bacilli spread throughout the brain substance, meninges and adjacent tissues, causing the formation of multiple, small, granulomatous foci having different sizes and degrees of encapsulation which may coalesce to form larger caseous foci. If such foci are adjacent to the ependyma or pia mater they may eventually rupture into the subarachnoid space, causing meningitis (Leonard 2017).

CNS TB thus occurs in sustained post-primary bacillaemia conditions (as in malnourished children under 3 years of age) and those involving a lack of sustained immunological control of latent foci in the brain or other body regions (as in the elderly and other immunosuppressed or HIV-infected adults). Granulomatous focus rupture into the subarachnoid space triggers an intense cytokine-mediated inflammatory reaction that becomes more marked at the base of the brain, causing basal proliferative arachnoiditis, vasculitis or hydrocephalus. Direct mycobacterial invasion of the blood vessel wall, or secondary extension of adjacent inflammation, leads to an intense polymorphonuclear reaction within the tunica adventitia vascularis, followed by lymphocyte, plasma cell and Mφ infiltration. Progressive destruction of the adventitia enables the inflammatory process to reach the tunica intima vascular, causing aneurysms, thrombosis and haemorrhage (Dian et al. 2021)

The extension of the inflammatory process to the cisterns can impede the circulation and cerebrospinal fluid absorption, giving rise to a communicating hydrocephalus. Typical tuberculous meningitis symptoms are a progressive subacute febrile picture beginning with a prodrome of general malaise, weakness, intermittent headache, sometimes vague discomfort in the neck or back and changes in personality. Subsequently, prolonged headache, meningism, vomiting, mild confusion and various degrees of cranial nerve palsies and signs of ascending pathways are experienced. Severe phases may involve delirium followed by stupor and coma, seizures, multiple cranial nerve deficits, hemiparesis and hemiplegia, and even death. Like pleural TB, it is paucibacillary, so it is unusual to find *Mtb* in cerebrospinal fluid. Spinal fluid analysis usually reveals increased cellularity at the expense of lymphocytes, hyperproteinorrachia with glucose consumption and often elevated ADA. Early tuberculosis management and the concomitant use of steroids are the therapy of choice (Chin 2014; Leonard 2017; Dian et al. 2021).

The clinical spectrum includes intracranial tuberculomas, defined as 2–8 cm avascular granulomatous masses in the brain parenchyma that develop deeply following disseminated bacillaemia. There is caseation necrosis within them and the bacillus can be isolated in the material so obtained. Lesions usually coalesce to form caseous granulomas having fibrous encapsulation; however, if an immune response against the mycobacteria is poor, focal cerebritis and subsequent abscess formation may develop. The clinical picture is characterised by fever (usually low), weight loss, diaphoresis, headache, seizures, progressive hemiplegia and/or signs of increased intracranial pressure (Garg 1999; Leonard 2017; Dian et al. 2021).

The rupture of a focus within the spinal cord or meninges, or extension of an adjacent area of spondylitis, may be associated with the development of arachnoiditis or tuberculoma at any level of the spinal cord. The triggered inflammatory reaction is usually limited to the involved region and can gradually progress over weeks to months, leading to the appearance of a gelatinous or fibrous mass partially or totally covering the spinal cord. Clinical manifestations are associated with compression of the nerve root and spinal cord, secondary to impingement by advancing arachnoiditis. Clinical manifestations are predominantly neurological and include pain, hyperesthesia or paraesthesia in nerve root distribution, lower motor neuron palsy and loss of sphincter control (Leonard 2017; Dian et al. 2021).

#### Genitourinary tuberculosis

All urinary and reproductive tract tissues become infected during mycobacterial extrapulmonary seeding, occurring in 2–20% of cases. Any part of the tract may become involved; however, the most frequent involvement involves the kidneys, ureters, bladder, prostate and ovaries. The Mycobacterium’s slow replication rate, intracellular location in Mφ and acquired immune response mean that the symptoms and signs of infection take between 12 months and 2 years after primary infection for urogenital disease to manifest itself. Constant chronic interaction between the mycobacteria and host immune response can lead to eradication of the microorganism, or disease progression manifesting as caseous necrosis, miliary disease and the formation of abscesses, cysts, ulcers and fistulas, fibrosis or calcifications. Cell-mediated immune responses become reduced when immunosuppression occurs (HIV infection), enabling tubercle bacilli proliferation, causing a more severe clinical picture that progresses rapidly. Mycobacterial seeding occurs in various parts of the urogenital tract through haematogenous or lymphatic spread from a primary pulmonary or intestinal tuberculous focus (Zachoval et al. 2018). It has been suggested that mycobacteria spread in the urine from the kidney to the collecting system and to the pelvic-calyceal system and then flows distally (Figueiredo et al. 2017; Zachoval et al. 2018; Muneer et al. 2019). A large percentage of urogenital TB cases may remain subclinical (Zachoval et al. 2018).

Regarding renal TB, mycobacterial-induced granulomas and tissue granulation with caseous necrosis may occur in all renal tissues, mainly in the cortex in the region adjacent to the glomeruli or peritubular capillary beds. Renal involvement is diffuse in HIV-infected patients or in other cases of immunosuppression, granulomas are less formed and lymph nodes are affected by numerous tubercle bacilli. Granulomatous inflammation and disease progression lead to chronic tubulointerstitial nephritis, papillary necrosis, ulcers, fibrosis with extensive caseous destruction of the renal parenchyma and lobulation, calyceal dilation and cavitations. Its spread to the renal pelvis can cause tuberculous pyelonephritis which can progress to pyonephrosis with progressive fibrosis and scarring of the renal pelvis and ureteropelvic junction, leading to urinary flow obstruction and pyelocalyceal dilation.

Symptoms and signs are nonspecific; the disease progresses to destruction of the renal parenchyma without treatment and consequent obstructive nephropathy with end-stage renal failure (Shah et al. 2015; Krishnamoorthy 2017). Up to 50% of patients having renal involvement have ureteral involvement. The bacilli spread from medullary renal lesions with urine into the ureters, ureterovesical junction and bladder. Ureteral involvement leads to inflammation, oedema, granulomatous ulceration and fibrosis, with resultant irregular ureteral stricture, segmental dilatation, ureteral obstruction and reflux. Urethritis continues with mucosal involvement and granuloma formation in the thickness of the ureteral wall and chronic inflammation with associated ureteral strictures leading to progressive hydroureteronephrosis (Kulchavenya 2014; Muneer et al. 2019).

Bladder TB usually arises from the urinary spread of mycobacteria from the upper urinary tract; it occurs in up to 21% of patients suffering renal TB and can present as cystitis in which there is superficial granulomatous inflammation with oedema of the mucosal surface that can be focal or generalised (Muneer et al. 2019). Sterile pyuria in urinalysis is the classic finding suggestive of urinary TB; however, it requires confirmation. Beaded images of the ureters in excretory urography and the gallbladder have also been described. Urine smear microscopy is not useful because normal commensal mycobacteria of the urinary tract can falsify the result, which is *Mycobacterium smegmatis*; however, culture or molecular tests can confirm the diagnosis (Mert et al. 2020).

Prostatic infection by mycobacteria can be caused by haematogenous or lymphatic spread from a pulmonary, renal or local focus; it is thus common for it to coexist with renal TB or tuberculous epididymo-orchitis. Mycobacterial infection of the prostate leads to chronic inflammation and caseous necrosis with cavitation and abscess formation (Kulchavenya et al. 2014). TB-related scrotal involvement (testis, epididymis and vas deferens) may occur, usually being associated with an active pulmonary focus. TB of the seminal vesicles and vas deferens can occur secondary to prostatic involvement. Scrotal involvement is usually secondary to haematogenous or lymphatic spread of tubercle bacilli, or by contiguous spread from the urinary tract or prostate. Inflammatory reaction of the scrotal or seminal vesicles usually leads to the formation of tuberculomas, calculi, cavitations and abscesses. The inflammation can cause scarring and anatomical distortion of the spermatic pathway, causing infertility. Stenosis development in the vas deferens and ejaculatory ducts secondary to inflammation leads to the development of obstructive azoospermia (Das et al. 2016).

All female reproductive tract components can become affected, although the endometrium and the uterine tubes are the most affected places. The infection route is usually through mycobacterial haematogenous or lymphatic dissemination from a pulmonary focus. Female genital involvement by mycobacteria is a chronic disease and may remain subclinical; patients often present a combination of malaise, abdominal pain, pelvic pain, menstrual irregularity, amenorrhoea, vaginal discharge, postmenopausal bleeding or infertility (Sharma 2015; Grace et al. 2017; Yadav et al. 2017).

#### Spinal tuberculosis

Spinal tuberculosis is one of the oldest known diseases and is often referred to as Pott’s disease. Sir Percival Pott (1779) described paraplegia due to the destruction of the anterior vertebral column and progressive kyphosis. Pott’s disease is then defined as the presence of tuberculous spondylodiscitis that may or may not be accompanied by psoas abscess (Wong-Taylor et al. 2013). Spinal TB is the most common musculoskeletal manifestation, affecting around 1–2% of all TB cases, 50% of musculoskeletal TB and 8% of all TB cases (Dunn and Ben Husien 2018; Jain et al. 2020). Spinal TB follows *Mtb’s* haematogenous dissemination route in the cancellous bone of the vertebral bodies from pulmonary lesions or the genitourinary system through venous or arterial routes. Haematogenous spread is facilitated by the arterial arcade that flows through each vertebra’s subchondral region, from the anterior and posterior spinal arteries forming a rich local vascular plexus. The disease is characterised by the paradiscal destruction of a vertebral body leading to kyphosis with preservation of the intervertebral disc until disease progresses. Kyphosis with epidural pus and disc and bone debris can lead to spinal cord compression and neurologic sequelae. Late-onset paraplegia can occur, despite resolution of active disease, due to persistent severe kyphosis, leading to irreversible myelomalacia and poor neurological recovery despite surgery (Jain et al. 2020).

Spinal onset is insidious; weight loss is the most consistent constitutional symptom, though fatigue, fever, night sweats and generalised pain may also occur. It usually presents with axial pain in the affected region having variable intensity that can become disabling. There may be specific symptoms according to the involvement of the different regions of the spine. Neurological deficit is common and occurs in 23–76% of cases, having higher prevalence when cervical and thoracic regions are involved. The detection of suspected symptoms is the most important element for diagnosing this condition; complementary tests will be necessary to confirm the existence of the disease, such as diagnostic images and microbiological studies. Treatment is mainly based on therapy with anti-TB drugs, analgesics and possible surgical treatment (mainly to treat complications) (Dunn and Ben Husien 2018; Jain et al. 2020).

#### Intestinal tuberculosis

It has been found that 10% of all extrapulmonary TB cases are intestinal. Intestinal TB has a poor prognosis, especially if there are life-threatening complications, such as intestinal stricture, obstruction, perforation and bleeding. The ileocecal region is the most affected region. It can spread from a tuberculous focus in another location, by haematogenous spread, or from an adjacent affected organ, such as the kidneys. *Mtb* causes an inflammatory reaction once it colonises the intestinal wall and the formation of granulomas leading to caseous necrosis and intestinal ulcers which are complicated by spontaneous healing and fibrosis. It is paucibacillary in nature, so *Mtb* culture is difficult, making the risk of false negatives very likely (Kedia et al. 2019; Maulahela et al. 2022). Milk was frequently contaminated with *Mycobacterium bovis* before pasteurisation became part of our lives, i.e. the cause of most intestinal TB. The two pathogens are very similar in structure and genetic identity, sharing 95% of their DNA, although both maintain remarkable selectivity for the species they affect. *Mycobacterium bovis* may affect humans, giving rise to intestinal TB (Moule and Cirillo 2020).

Its clinical manifestations are not precise and can mimic a variety of other diseases, the commonest being chronic or acute abdominal pain if acute complications occur, such as weight loss, anaemia, fever, night sweats, chronic diarrhoea, constipation and decreased appetite. Intestinal bleeding, fistulas and intestinal perforation with associated tuberculous peritonitis are the most often found complications (Shi et al. 2016; Patel and Yagnik 2018). The symptoms, history and diagnostic images (computerised axial tomography (CT) and nuclear magnetic resonance (NMR)) can assist diagnosis, but precise confirmation must be made with endoscopy and biopsy for microbiological study, culture and specific molecular tests. Treatment consists of administering anti-tuberculosis drugs and surgical management must be resorted to when there are abdominal complications (Maulahela et al. 2022).

#### Other presentations of tuberculosis

Pericardial TB presents pericardial effusion which can compromise cardiac function; effusion is usually a predominantly monocytic exudate, with positive ADA (Ramírez-Lapausa et al. 2015). Management may require steroids to decrease the likelihood of constrictive pericarditis, especially in HIV patients. This, however, is still a matter of debate (Ntsekhe et al. 2003). Cutaneous TB is a rare presentation, often accompanied by pulmonary TB; this presents skin lesions due to local inoculation of mycobacteria or by haematogenous spread. Diagnosis is confirmed by biopsy (Ramírez-Lapausa et al. 2015). Another less frequently occurring presentation is TB of the joints but which may be clinically relevant; similar to the meningeal or pleural forms, it is paucibacillary. TB’s clinical manifestations are variable, depending on a patient’s immune status, the dissemination route and the affected organ. This means that TB can be considered the “great simulator” since it can resemble practically any disease. TB diagnosis requires strong clinical suspicion and obtaining samples for microbiological and molecular analysis.

### Current perspectives regarding TB vaccines

There is an urgent need for developing low-cost and more effective TB vaccines, given the burden of disease and mortality associated with TB and a steadily increasing global burden concerning TB resistance. Although new antibiotic regimens are being developed, there is an urgent need for developing low-cost TB vaccines having increased efficacy. This is accompanied by concerns that currently available financial resources for testing TB vaccines in a clinical setting are extremely low, despite the disease’s global burden (World Health Organization 2022). TB vaccines have relied on a CD4 + Th1 TL response for immunogenicity stage determination of candidate vaccines; however, the full protection mechanism is not yet well-understood and it has been suggested that IFN-γ production by this cellular subset alone is neither sufficient nor fully predictive of clinical vaccine efficacy (Lu et al. 2019). Although CD8 + T-cell importance regarding TB has been emphasised, they have not been given sufficient importance to be considered as target cells when developing vaccine candidates (Martin et al. 2020; Lewinsohn et al. 2017).

**Fig. 5:**
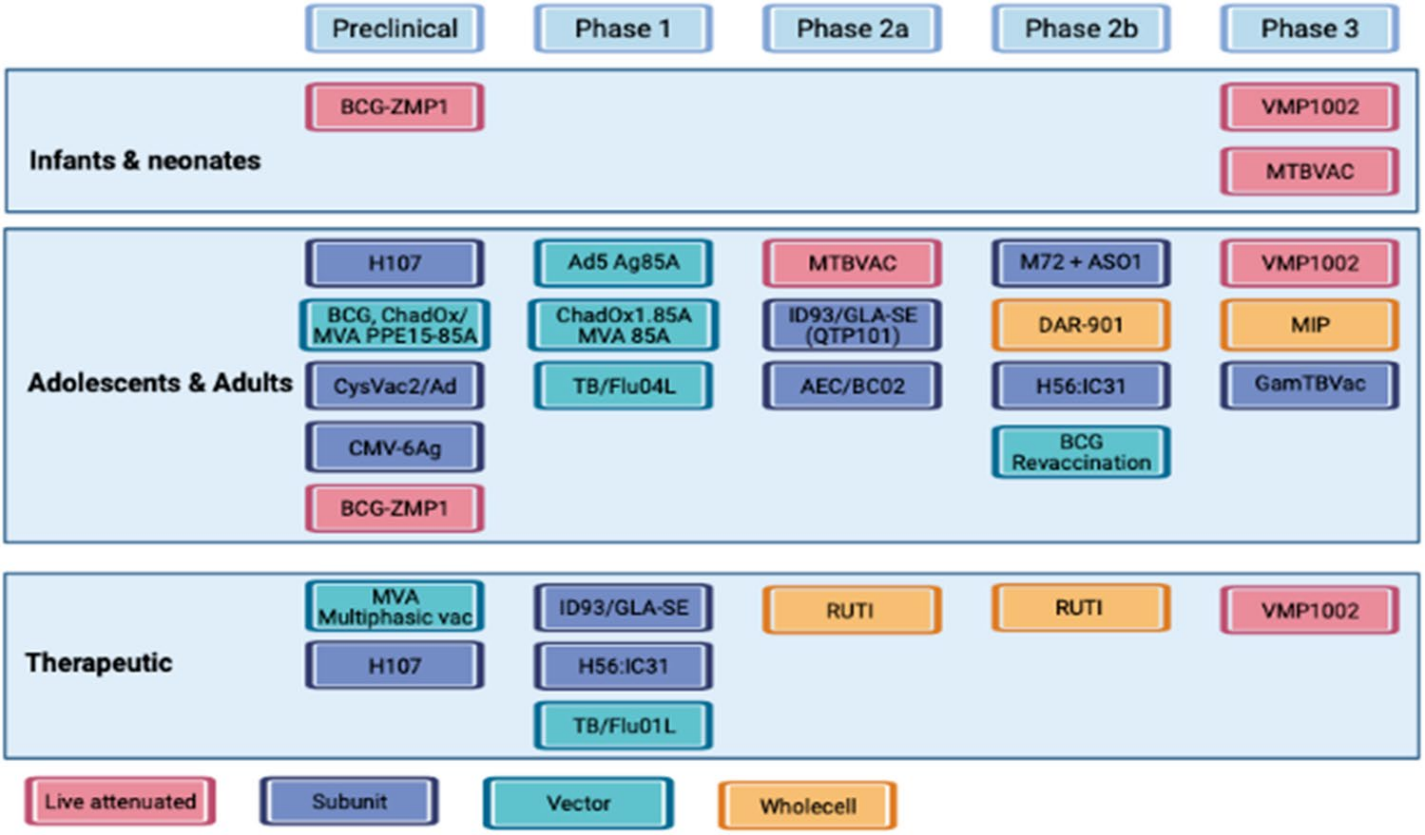
Tuberculosis antigens in clinical trials. Summary of TB vaccine candidates currently in clinical trials (last update October 2022) (https://www.tbvi. eu/what-we-do/pipeline- vaccines/, accessed on 19th April 2023)

The TuBerculosis Vaccine Initiative (TBVI) is a research and innovation partnership that facilitates TB vaccine discovery and development and works through the Global TB Vaccine Partnership (GTBVP). According to TBVI data (https://www.tbvi. eu/what-we-do/pipeline- vaccines/, accessed on April 19th, 2023), the current TB vaccine pipeline includes 29 candidate vaccines in different stages of clinical development (Fig. 5), belonging to different categories: live attenuated mycobacterial vaccines, killed and fragmented whole-cell vaccines for therapeutic purposes, adjuvanted protein subunit vaccines based on one or more recombinant *Mtb* fusion proteins and viral vector vaccines. Improving the efficacy of prophylactic anti-*Mtb* vaccines and increased investment to cover manufacturing input costs are therefore considered a necessity to ensure that a vaccine is readily available for those who need it. There has been increasing enthusiasm regarding current TB vaccine research, along with good progress concerning preclinical and clinical trials (Romano et al. 2023).

New strategies have emerged concerning vaccine design with advances made regarding materials science, thereby enabling precise delivery, enhanced adjuvant function, greater sparing effect, increased stabilisation and gradual release at induction site (Ali et al. 2023). Nanoparticles (NPs) have been used for antigen transport along with their adjuvant characteristics and a new TB vaccine called Nano-FP1 has recently been created using yellow carnauba wax NPs coated with a fusion protein containing three *Mtb* antigens (Acr, Ag85B and HBHA), which has had good results in the preclinical phase (Hart et al. 2018).mRNA technology has progressed over the years and a leap forward was made during the COVID-19 pandemic; the time is thus right for using this technology for developing a new and effective TB vaccine. Exploring TB-related mRNA technology is not new, as a 2004 study by UK researchers showed that an mRNA vaccine offered modest but significant protection against *Mtb* in mice (Xue et al. 2004).

Nine proteins were exploited in a recent study for predicting epitopes aimed at developing an mRNA vaccine against TB; many immunoinformatics tools were used for constructing this vaccine to elicit cellular and humoral immunity. Thirty epitopes, an adjuvant TLR4 agonist RpfE, subcellular trafficking constructs, secretion booster and specific linkers were combined for developing the vaccine. Such proposed construct was predicted to cover 99.38% of the population when tested; it was tested to be effective and safe. An in silico immune simulation of the vaccine was also performed to validate the base hypothesis. The vaccine peptide’s secondary and tertiary structures were predicted and docked against TLR-4 and TLR-3. Molecular dynamics simulation validated binding complex stability. Such proposed construct can thus be considered a promising anti-TB vaccine; the researchers proposed that their construct is ready for wet-lab experiments for its efficacy to become approved (Al Tbeishat 2022).

A second-generation TB vaccine candidate ID91 was produced in another recent study as a fusion protein formulated with a synthetic TLR4 agonist (glucopyranosyl lipid adjuvant in a stable emulsion) or as a novel replicating-RNA (repRNA) formulated in a nanostructured lipid carrier. Protein subunit- and RNA-based vaccines preferentially elicit cellular immune responses targeting different ID91 epitopes; both platforms reduced pulmonary bacterial burden in a single prophylactic immunisation screen, compared to controls. Excitingly, prime-boost strategy groups that received heterologous RNA-prime, protein-boost or combination immunisations had the greatest reduction in bacterial burden and a unique humoral and cellular immune response profile. Such data represents the first report that repRNA platforms are a viable system for TB vaccines and should be pursued using high-priority *Mtb* antigens containing CD4 + and CD8 + T-cell epitopes (Larsen et al. 2023).

An mRNA-based vaccine can thus be developed for preventing TB; however, in practice this will depend on identifying the most appropriate antigen targets (which has been difficult to achieve to date). The WHO’s new mRNA technology transfer hubs have raised researchers’ optimism that the time has come to find a new anti-TB vaccine. The WHO experts consider that mRNA technology transfer hubs would empower low- and middle-income countries to produce their own vaccines, medicines and diagnostics, and the technology could also be used for developing TB vaccines. The mRNA technology has progressed over the years and actually leap-frogged during the COVID-19 pandemic; it is thus the right time to use mRNA technology for developing a new and effective TB vaccine.

## Conclusions

TB is the oldest documented infectious disease in humans, infecting almost a third of the world’s population. The host-microbe interaction is responsible for a broad clinical and pathological spectrum. Understanding TB immunology is essential for developing new diagnostic and therapeutic tools and forms the basis for developing an effective anti-TB vaccine.

Research into this disease facilitates further understanding of the mycobacteria’s complex nature as it affects the usual host cell functions. It can directly inhibit the main immune system, as seen above; host defence system has a double-branched arsenal against mycobacteria, i.e. innate and adaptive immunity. Taken together, this leads to pathogen elimination and preventing disease development. Trained immunity should not be ignored as it also seems to be responsible for humans’ partial protection against mycobacteria.

The host immune system employs a broad spectrum of mechanisms to stop tubercle bacillus advance and pathogenesis. However, the pathogen has developed its own defence system, directly focusing on evading our immune response and thereby leading to the development of the clinical manifestations described in this review. Granuloma formation may control *Mtb* growth, although it is now thought that mycobacteria may hijack granulomas for their own advantage, thereby altering the immune response and enabling their dissemination via haematogenous, bronchogenic and lymphatic pathways from an initial pulmonary focus.

*Mtb* induces a new acute local immune response upon reaching the different organs and tissues after its dissemination; this could become chronic and its cellular inflammatory changes and cytokine and inflammation mediator production will cause the histopathological changes and systemic effects which are often not organ-specific, making TB the “great clinical simulator”. Clinical suspicion is thus highly relevant when arriving at diagnostic suspicion regarding the certainty of TB in its many forms, followed by timely establishment of suitable treatment, as necessary, according to host organs compromised by the tuberculosis bacillus.

TB continues to be one of the twenty-first century’s most important public health problems; clinical and scientific advances have increasingly led to greater preventive, pathophysiological, immunological, diagnostic and therapeutic knowledge regarding all forms and clinical presentations of this disease. Collaborative multinational efforts must be continued to have an ever deeper and clearer conceptual understanding of TB that will enable it to be eradicated. Such collaborative efforts should include researchers, public health workers, health care staff and the general population with the sole purpose of expanding current knowledge and mitigating TB’s global burden.

## Data Availability

The authors confirm that all relevant data are included in this article.
